# Antibiotics and the Endocrine System: Mechanisms, Adverse Effects, and Clinical Implications

**DOI:** 10.3390/antibiotics15090857

**Published:** 2026-09-02

**Authors:** Łukasz Grabarczyk, Julia Oszytko, Maria Derkaczew, Kamil Sobolewski, Paweł Radkowski

**Affiliations:** 1 Alarm Clock Clinic, Coma Recovery and Neurorehabilitation Center, 04-730 Warsaw, Poland; l.grabarczyk@budzikdladoroslych.pl; 2 Faculty of Medicine, Opole University, 45-758 Opole, Poland; 136934@student.uni.opole.pl; 3 Department of Physiology and Pathophysiology, Faculty of Medicine, Collegium Medicum, University of Warmia and Mazury, Al. Warszawska 30, 10-082 Olsztyn, Poland; 4 Clinical Department of Anaesthesiology and Intensive Care, Regional Specialist Hospital in Olsztyn, 10-561 Olsztyn, Poland; kamil.roman.sobolewski@gmail.com; 5 Department of Anaesthesiology and Intensive Care, School of Medicine, Collegium Medicum, University of Warmia and Mazury in Olsztyn, 10-719 Olsztyn, Poland; 6 Hospital zum Heiligen Geist, 34560 Fritzlar, Germany

**Keywords:** antibiotics, endocrine adverse effects, hypoglycemia, hyponatremia, SIADH, endocrine disruption, intensive care

## Abstract

Background/Objectives: Antibiotics are widely used in clinical practice, yet their potential effects on endocrine homeostasis remain insufficiently recognized. This review summarizes endocrine adverse effects associated with selected antibacterial agents, their proposed mechanisms, and clinical relevance. Methods: A narrative literature search was conducted in PubMed/MEDLINE, Scopus, and Web of Science from database inception to 10 August 2026. Clinical, pharmacovigilance, case-based, animal, in vitro, and ex vivo studies were considered. Results: Reported disturbances include SIADH-related hyponatremia, hypoglycemia, altered thyroid hormone metabolism, changes in hypothalamic–pituitary–adrenal axis activity, aldosterone dysregulation, and reproductive hormone disturbances. Selected cephalosporins and carbapenems have been associated with changes in prolactin secretion, thyroid-related parameters, and gonadal function. Fluoroquinolones have been associated with dysglycemia, while effects on reproductive hormone pathways have been reported predominantly in experimental models. Selected macrolides may influence glucocorticoid metabolism and KCNJ5-dependent aldosterone production. Linezolid has been linked to hyponatremia and hypoglycemia, while metronidazole may affect pituitary hormone secretion. Trimethoprim–sulfamethoxazole may contribute to hyponatremia, hyperkalemia, hypoglycemia, and thyroid dysfunction. The supporting evidence is heterogeneous and ranges from case reports and pharmacovigilance analyses to experimental studies. Human clinical evidence is strongest for selected disturbances of glucose and electrolyte homeostasis, whereas many reported reproductive, pituitary, thyroid, and adrenal effects remain supported predominantly by experimental or case-based evidence. Conclusions: Many proposed mechanisms remain incompletely established and cannot be directly extrapolated to routine practice. Awareness of these complications may support earlier recognition, targeted biochemical monitoring, and safer antibiotic therapy, particularly in older, critically ill, or renally impaired patients and those exposed to polypharmacy.

## 1. Introduction

Antibiotics are among the most widely used drug classes in medicine. Their consumption, according to available data, increased by 16.3% from 2016 to 2023, reaching 34.3 billion defined daily doses [1]. The widespread use of antibiotics has been accompanied by increasing recognition of their adverse drug reactions (ADRs). Frequently reported ADRs include allergic and hypersensitivity reactions, as well as neurological and gastrointestinal adverse effects [2].

The endocrine system is a complex regulatory network essential for maintaining physiological homeostasis. Endocrine disturbances associated with antibacterial therapy are infrequently reported but may occasionally be clinically significant. Proposed mechanisms include direct effects on endocrine cells, altered neuronal signaling, microbiota-related changes, and modulation of enzymes involved in hormone synthesis. Syndrome of inappropriate antidiuretic hormone secretion (SIADH) has been reported in association with several antibacterial agents, including nitrofurantoin, trimethoprim–sulfamethoxazole, linezolid, azithromycin, and rifabutin [3,4,5,6,7,8]. In a murine model, administration of a broad-spectrum antimicrobial regimen consisting of ampicillin, vancomycin, neomycin, metronidazole, and amphotericin B was associated with reduced vasopressin expression in the adult brain. These findings suggest that antimicrobial-induced microbiota depletion may influence central neuroendocrine signaling [9,10]. This represents a putative indirect, microbiota-mediated pathway rather than evidence of direct endocrine toxicity.

Antibiotic-associated hypoglycemia has been reported in both patients with and without diabetes, although the risk varies according to the antibacterial agent and individual clinical factors. Older age, renal impairment, concomitant glucose-lowering therapy, and polypharmacy may increase susceptibility to this complication. Several antibacterial agents, including cefditoren, clarithromycin, ertapenem, moxifloxacin, tigecycline, levofloxacin, and linezolid, have been associated with hypoglycemic events [11].

Research increasingly recognizes the importance of gut microbiota in modulating brain function [12,13,14,15] through neural, immune, metabolic, and endocrine pathways [16]. The HPA axis is sensitive to stress-related and developmental influences [17,18,19,20,21,22], while experimental studies suggest that gut microbial composition can also modulate HPA-axis regulation [13,23]. A study in germ-free mice demonstrated an exaggerated hypothalamic–pituitary–adrenal axis (HPA-axis) response to restraint stress, characterized by greater increases in plasma adrenocorticotropic hormone (ACTH) and corticosterone than in specific pathogen-free controls. This response was reversed by colonization with *Bifidobacterium infantis* and partially corrected by early, but not late, reconstitution with specific pathogen-free microbiota [24]. These findings raise the possibility that antibiotic-induced dysbiosis may alter HPA-axis regulation, although direct clinical evidence remains limited.

Changes in gut microbial composition have also been associated with thyroid-related pathways. The microbiota interacts with the thyroid gland through the gut–thyroid axis, playing an important role in the pathogenesis of autoimmune thyroid disorders [15,25]. Furthermore, bacterial metabolites, including secondary bile acids and short-chain fatty acids, also modulate the function of the axis [12]. Disturbances in the microbial balance can cause damage to the intestinal barrier and subsequent exposure to bacterial antigens. Cross-reactivity can destabilize T cell subpopulations, leading to an inflammatory response within the thyroid gland [25].

Data regarding the effects of antibiotics used in intensive care units and anesthesiology on the endocrine system remain very limited. Their clinical implications are largely based on studies in animal models or cell cultures, and their mechanisms are not fully understood. For the purposes of this review, these findings are distinguished according to their likely basis as direct or putative endocrine effects, pharmacokinetic drug interactions, renal or metabolic effects producing endocrine-like abnormalities, microbiota-mediated mechanisms, or experimental endocrine effects with potential therapeutic relevance. This narrative review aims to summarize and critically discuss the available evidence on endocrine and metabolic effects associated with selected antibacterial agents, with particular emphasis on compounds for which clinically relevant observations or mechanistic data have been reported.

## 2. Beta-Lactams

Beta-lactam antibiotics are characterized by the presence of a beta-lactam ring and exert bactericidal activity primarily through inhibition of bacterial cell-wall synthesis by binding to penicillin-binding proteins. Their pharmacokinetic properties differ between individual agents and subclasses, although renal elimination is important for many beta-lactams and may substantially influence systemic drug exposure in patients with impaired kidney function [26,27]. The major beta-lactam classes include penicillins, cephalosporins, carbapenems, and monobactams. In the context of endocrine effects, the available evidence remains limited and concerns mainly selected cephalosporins and carbapenems.

### 2.1. Cephalosporins

Evidence regarding endocrine effects of cephalosporins is heterogeneous and is derived mainly from isolated case reports and experimental studies. Reported observations include hyperprolactinemia, alterations in thyroid hormone metabolism, hypoglycemia, and changes in testosterone levels. Two case reports have described suspected cefpodoxime-associated hyperprolactinemia with galactorrhea. In one patient, symptoms developed after two days of cefpodoxime treatment at a dose of 200 mg twice daily and resolved after discontinuation, with normalization of serum prolactin levels within one week [28]. In the second case, similar symptoms occurred after 14 days of treatment and resolved after cefpodoxime withdrawal; however, concomitant venlafaxine use represents a potential confounding factor because this drug may itself be associated with hyperprolactinemia and galactorrhea [29].

In a culture-based experiment, cefuroxime reduced endogenous T3 levels, an effect attributed to inhibition of type 2 deiodinase (D2). Ceftazidime is another drug that inhibits this enzyme without affecting D1 and D3 deiodinase activity. These enzymes play a key role in the conversion of T4 to T3 in tissues. In an animal model, cefuroxime administration was associated with moderate changes, manifested by a reduction in the diameter of thyroid follicles. Another effect of this antibiotic was disruption of the thyroid–pituitary axis due to reduced pituitary sensitivity to T4/T3 [30].

In an analysis of the FDA Adverse Event Reporting System, cefditoren showed a strong reporting association with hypoglycemia [11]. Cefditoren pivoxil contains a pivalate moiety, and prolonged exposure to pivalate-containing antibiotics may reduce carnitine availability. Carnitine depletion can impair fatty acid oxidation and gluconeogenesis, thereby increasing susceptibility to hypoglycemia, particularly in patients with limited metabolic reserves [31,32].

Continuous cefuroxime administration in an animal model was associated with a reversible decrease in testosterone [33].

### 2.2. Carbapenems

Clinical evidence regarding endocrine adverse effects of carbapenems is very limited. Experimental studies, however, have suggested potential effects on male reproductive function. In an animal model, imipenem exposure was associated with testicular toxicity and reduced serum testosterone levels [33,34].

## 3. Fluoroquinolones

Fluoroquinolones are synthetic bactericidal agents that inhibit bacterial DNA gyrase and topoisomerase IV, thereby interfering with DNA replication and chromosome integrity. They generally demonstrate moderate-to-high oral bioavailability and extensive tissue distribution, although elimination differs considerably between individual agents, ranging from predominantly renal excretion to substantial hepatic metabolism. These pharmacokinetic differences may be clinically relevant when considering adverse effects and drug interactions, particularly in older patients and those with renal impairment [35,36].

Rare cases of ciprofloxacin-associated SIADH have been reported. The mechanism of ciprofloxacin-associated SIADH remains uncertain. Because fluoroquinolones can cross the blood–brain barrier and interact with GABAergic signaling, modulation of GABA receptors has been proposed as one possible mechanism affecting ADH secretion [2,37,38,39,40]. Reported cases have predominantly involved older adults, with symptoms generally resolving within several days after ciprofloxacin discontinuation [37,38].

Ciprofloxacin may reduce the gastrointestinal absorption of levothyroxine, representing a clinically relevant drug interaction. Evidence that ciprofloxacin directly alters thyroid hormone synthesis or expression is lacking [41]. Fluoroquinolones have been associated with dysglycemia, including hypoglycemia, although the magnitude of this risk varies among individual agents [11,42]. Experimental data suggest that some fluoroquinolones may stimulate insulin secretion by inhibiting ATP-sensitive potassium (KATP) channels in pancreatic β-cells; however, this mechanism appears to be agent-dependent and should not be extrapolated uniformly to the entire class [43].

The clinical relevance of this association is supported by regulatory and observational evidence. In 2018, the U.S. Food and Drug Administration strengthened warnings for systemic fluoroquinolones regarding serious hypoglycemia, including hypoglycemic coma, particularly in older patients and in patients with diabetes receiving glucose-lowering medications [44]. Observational studies have likewise identified dysglycemic events during fluoroquinolone therapy, with concomitant sulfonylurea treatment representing an important risk factor [45]. Pharmacovigilance analyses have further identified signals for hypoglycemia associated with individual fluoroquinolones; however, spontaneous-reporting data do not permit estimation of incidence or establish causality [11].

Experimental studies suggest that selected fluoroquinolones may alter reproductive and thyroid hormone profiles. Norfloxacin exposure in juvenile common carp was associated with changes in testosterone, progesterone, and thyroid hormone concentrations [46]. Other experimental studies have reported reductions in FSH, LH, and testosterone following ciprofloxacin or enrofloxacin exposure [47]. These findings remain experimental, and their relevance to standard therapeutic exposure in humans is uncertain.

## 4. Macrolides

Clarithromycin may increase the risk of hypoglycemia through clinically relevant interactions with glucose-lowering agents. Concomitant administration of clarithromycin and repaglinide has been associated with severe hypoglycemia. Clarithromycin inhibits CYP3A4 and has been shown to increase plasma repaglinide concentrations and its glucose-lowering effects [48,49]. Severe hypoglycemia has also been reported during concomitant treatment with clarithromycin and sulfonylureas [50]. In addition to these drug–drug interactions, an analysis of the FDA Adverse Event Reporting System identified a signal for hypoglycemia associated with clarithromycin even in reports without concomitant sulfonylurea or meglitinide use [11]. However, because spontaneous reporting databases cannot establish incidence or causality, these findings should be interpreted cautiously.

Experimental data suggest that macrolides may modulate glucocorticoid metabolism and HPA-axis activity. Park et al. reported modulation of 11β-HSD expression associated with clarithromycin and azithromycin in sinonasal epithelial models [51]. In a separate experimental study, Yamamoto et al. observed increased endogenous corticosterone levels following roxithromycin administration in mice [52]. These findings remain experimental and should not be directly extrapolated to routine clinical antibiotic therapy.

Somatic mutations in the KCNJ5 potassium channel, particularly G151R and L168R, are commonly identified in aldosterone-producing adenomas. These mutations impair potassium selectivity, resulting in increased sodium influx, membrane depolarization, calcium entry, and enhanced aldosterone synthesis. In vitro studies demonstrated that selected macrolides, including roxithromycin and clarithromycin, selectively inhibited mutant KCNJ5 channels, with little or no effect on wild-type KCNJ5. Roxithromycin also reduced CYP11B2 expression and aldosterone production in human adrenal-derived cell lines expressing mutant KCNJ5 [53].

These findings were subsequently supported by an ex vivo study in which clarithromycin reduced CYP11B2 expression and aldosterone secretion in aldosterone-producing cells isolated from human adenomas carrying the G151R or L168R KCNJ5 mutations, but not in cells obtained from adenomas without these mutations [54]. These observations suggest that macrolide-derived compounds may have potential diagnostic or therapeutic applications in patients with KCNJ5-mutated aldosterone-producing adenomas. Accordingly, this finding should be considered a potential therapeutic endocrine effect rather than an adverse endocrine reaction to macrolide therapy. However, this concept remains experimental and requires confirmation in clinical studies.

A clinical proof-of-concept study was subsequently designed to investigate whether clarithromycin and roxithromycin could alter aldosterone secretion in patients with KCNJ5-mutated aldosterone-producing adenomas [55].

The potential influence of clarithromycin on reproductive hormone homeostasis has been investigated primarily in the context of pharmacokinetic interactions. In one animal study, male mice received quinestrol alone or quinestrol combined with increasing doses of clarithromycin. Quinestrol administered alone and in combination with clarithromycin reduced serum testosterone and luteinizing hormone concentrations compared with the control group. The addition of higher clarithromycin doses was associated with greater suppression of selected reproductive parameters, particularly serum LH levels and sperm density [56].

The authors proposed that clarithromycin, through inhibition of CYP3A4-mediated metabolism, may potentiate the antifertility effects of quinestrol. These findings therefore indicate a possible drug–drug interaction rather than a direct endocrine or gonadotoxic effect of clarithromycin. Moreover, because the study was conducted in mice using quinestrol as an experimental fertility-control agent, its relevance to standard clinical antibiotic therapy remains uncertain [56].

## 5. Linezolid

Linezolid-associated endocrine and metabolic abnormalities discussed in this review include hyponatremia/SIADH and hypoglycemia. Hyponatremia and SIADH have been reported during linezolid therapy in case reports, retrospective analyses, and pharmacovigilance data [4,6,57,58]. The underlying mechanism remains uncertain, although direct stimulation of ADH release has been proposed [4,6]. In one retrospective analysis, mild hyponatremia was reported in 18% of patients receiving linezolid, while severe hyponatremia was substantially less frequent [58]. Because clinically significant hyponatremia may occur, serum sodium monitoring should be considered in patients at increased risk.

Linezolid-associated hypoglycemia has been reported, particularly in patients with diabetes receiving insulin or oral glucose-lowering agents. Analysis of post-marketing reports identified a safety signal for hypoglycemia associated with linezolid therapy [59]. Linezolid is a reversible, non-selective monoamine oxidase (MAO) inhibitor, and MAO inhibition has been proposed as one possible mechanism contributing to this adverse effect. However, the causal relationship and exact pathophysiological mechanism remain incompletely established. Mitochondrial toxicity has also been described during prolonged linezolid exposure, although its direct contribution to hypoglycemia remains uncertain [60,61]. Therefore, blood glucose monitoring should be considered in patients at increased risk of hypoglycemia during linezolid therapy.

## 6. Metronidazole

Experimental evidence suggests that metronidazole and related nitroimidazole compounds may affect anterior pituitary function, although the direction of these effects differs between experimental models. In zebrafish larvae, metronidazole increased proopiomelanocortin (POMC) expression and was also associated with increased prolactin expression and proliferation of POMC-expressing pituitary cells [62]. In contrast, Stalla et al. demonstrated in cultured rat anterior pituitary cells that nitroimidazole derivatives inhibited ACTH, GH, and PRL secretion, apparently through an effect on the catalytic component of the adenylate cyclase system [63]. Histological alterations of pituitary tissue have also been reported in rats exposed to metronidazole. These divergent findings indicate that the pituitary effects of metronidazole are model-dependent and remain predominantly experimental, with uncertain clinical relevance in humans [64].

Experimental animal studies have reported reductions in serum testosterone and FSH following metronidazole exposure [65], with one study also reporting reduced LH concentrations [66]. The mechanism underlying these observations remains unclear.

## 7. Trimethoprim–Sulfamethoxazole

Trimethoprim–sulfamethoxazole has been associated with hyponatremia, including reported cases attributed to SIADH [7,67,68]. However, hyponatremia during TMP-SMX therapy may also result from the renal effects of trimethoprim and should not automatically be interpreted as SIADH. The renal mechanism is discussed below.

Animal studies suggest that prolonged or high-dose exposure to trimethoprim–sulfamethoxazole may impair thyroid function. In euthyroid dogs treated with co-trimoxazole for 21 days, serum T4 concentrations decreased in all treated groups, whereas a significant increase in TSH was observed only in the group receiving the highest dose. Treatment was also associated with increased relative thyroid weight and histopathological changes, including follicular cell hypertrophy and hyperplasia, severe colloid depletion, and intrafollicular hemorrhage [69]. A dose-dependent effect was also demonstrated in mice receiving commercial TMP-SMX-containing feed. A high daily SMX dose of 2400 mg/kg resulted in a significant reduction in plasma T4, an increase in TSH, and thyroid follicular hypertrophy and hyperplasia. In contrast, no significant changes in T4 were observed in mice receiving the lower SMX dose of 240 mg/kg/day. These findings indicate that the thyroid effects of TMP-SMX observed in animal models depend strongly on the dose and duration of exposure, and their clinical relevance to standard human therapy remains uncertain [70].

Hypoglycemia is a rare but potentially serious adverse effect of TMP-SMX and has been proposed to result, at least in part, from the sulfonylurea-like properties of sulfamethoxazole. In an analysis of 34 reported patients with TMP-SMX-associated hypoglycemia, the median age was 64 years, and 75.8% had renal dysfunction. Elevated serum insulin concentrations were reported in 15 patients (44.1%), while elevated C-peptide concentrations were observed in 13 patients (38.2%). The median duration of TMP-SMX treatment before the onset of hypoglycemia was six days, and hypoglycemic symptoms persisted from 8 h to 47 days after intervention. Renal impairment, high drug exposure, and malnutrition were identified as important predisposing factors [71].

Trimethoprim exerts an amiloride-like effect by inhibiting epithelial sodium channels in the distal nephron. Reduced sodium reabsorption decreases the electrochemical gradient required for potassium secretion and may result in hyperkalemia and a functional hypoaldosteronism-like state [72,73]. This effect primarily reflects direct renal tubular ENaC inhibition and should therefore be distinguished from primary adrenal or aldosterone deficiency.

The principal endocrine effects associated with the antibacterial agents discussed in this review, together with the proposed mechanisms and the type/source of supporting evidence, are summarized in Table 1.

## 8. Materials and Methods

This study was designed as a narrative review aimed at summarizing and critically discussing the reported endocrine and metabolic effects of selected antibacterial agents. A literature search was performed in PubMed/MEDLINE, Scopus, and Web of Science from database inception to 10 August 2026. The search focused on antibacterial agents and classes for which endocrine or metabolic effects had been reported and for which sufficient relevant literature was identified to permit meaningful narrative synthesis. Selection also considered their relevance to contemporary clinical practice, particularly in hospitalized and critically ill patients. The antibacterial search terms included beta-lactams, cephalosporins, carbapenems, fluoroquinolones, macrolides, linezolid, metronidazole, trimethoprim–sulfamethoxazole, and the names of individual agents discussed in the manuscript. Each antibacterial term was combined with endocrine-related terms, including “endocrine”, “hormone”, “pituitary”, “thyroid”, “adrenal”, “cortisol”, “aldosterone”, “SIADH”, “hyponatremia”, “hypoglycemia”, “insulin”, “testosterone”, “FSH”, “LH”, and “reproductive toxicity”. Reference lists of relevant publications were additionally reviewed to identify further potentially relevant studies.

Publications were considered for inclusion when they reported endocrine or metabolic adverse effects, endocrine-relevant drug interactions, or experimental mechanisms potentially linking antibacterial exposure with endocrine function. Given the limited and heterogeneous literature in this field, evidence from clinical studies, pharmacovigilance analyses, case reports, and case series, as well as animal, in vitro, and ex vivo studies, was considered. Publications without findings directly relevant to endocrine or metabolic outcomes were not retained for the narrative synthesis. Primary studies were preferred for specific mechanistic or safety claims where available, while review articles were used primarily to provide broader context and to identify additional primary sources.

The evidence was synthesized narratively according to the endocrine domain affected and the antibacterial agent involved. For interpretation of clinical relevance, human clinical and regulatory evidence was given greater weight than experimental evidence. Case reports and case series were retained because several of the adverse effects discussed are rare and are currently supported mainly by individual clinical observations. Animal, in vitro, and ex vivo studies were used primarily to discuss possible biological mechanisms and were not considered equivalent to evidence of clinical effects in humans. Pharmacovigilance findings were interpreted as safety signals and not as estimates of incidence or proof of causality.

No formal risk-of-bias tool or quantitative evidence-grading system was applied because of the narrative design and the substantial heterogeneity of the included evidence. Similarly, no meta-analysis was performed.

## 9. Discussion

The evidence reviewed in this article is highly uneven, both in terms of study design and clinical relevance. Among the reported endocrine complications, disturbances of glucose and electrolyte homeostasis are supported by the most direct human data. Fluoroquinolone-associated dysglycemia has been recognized in regulatory communications and observational studies, with older age, diabetes, concomitant glucose-lowering therapy, and impaired renal function emerging as clinically relevant risk factors [11,44,45]. Hypoglycemia during clarithromycin treatment is particularly relevant when the drug is combined with repaglinide or sulfonylureas [48,49,50], whereas trimethoprim–sulfamethoxazole-associated hypoglycemia appears to occur predominantly in susceptible patients, especially those with renal impairment [71]. Human observations also support an association between linezolid treatment and both hyponatremia and hypoglycemia [4,6,57,58,59]. By comparison, several proposed effects involving the pituitary, thyroid, or gonadal axes are still supported mainly by isolated reports or experimental models, which substantially limits their immediate clinical interpretation.

The mechanisms described across antibacterial agents are equally diverse and argue against treating endocrine adverse effects as uniform class properties. Some are best explained by drug–drug interactions. Clarithromycin can increase exposure to glucose-lowering drugs through CYP3A4 inhibition [49], while ciprofloxacin may interfere with gastrointestinal absorption of levothyroxine [41]. Other effects are linked to renal or metabolic pathways. Trimethoprim inhibits ENaC in the distal nephron and thereby reduces potassium secretion [73], whereas exposure to pivalate-containing antibiotics may reduce carnitine availability and contribute to hypoglycemia under susceptible metabolic conditions [31,32]. Experimental work suggests further mechanisms involving ion channels, including modulation of pancreatic KATP channels by some fluoroquinolones [43] and selective inhibition of mutant KCNJ5 channels by certain macrolides [53,54]. Taken together, these findings support interpretation at the level of the individual antibacterial agent and the specific biological pathway involved, rather than assuming a shared endocrine effect across an entire drug class.

Interpretation is particularly difficult in hospitalized and critically ill patients, in whom many of the same biochemical abnormalities may arise independently of antibacterial treatment. Acute infection, systemic inflammation, renal dysfunction, altered nutritional intake, concurrent medication, and pre-existing endocrine disease can all modify glucose, sodium, potassium, and hormone concentrations. In this setting, temporal association alone may be insufficient to establish a drug-related mechanism. The cefpodoxime reports illustrate this difficulty, because venlafaxine use in one patient represents an alternative explanation for hyperprolactinemia and galactorrhea [29]. Similar caution applies to pharmacovigilance databases: disproportionate reporting may identify a potential safety problem, but it does not establish its incidence or prove that the suspected drug was responsible. Experimental findings require a different form of caution, since species, administered doses, treatment duration, and experimental conditions may differ considerably from routine human exposure.

From a practical perspective, the available evidence supports a risk-based rather than routine approach to endocrine monitoring during antibacterial therapy. Particular attention may be warranted in older patients, individuals with renal impairment or pre-existing endocrine disease, critically ill patients, and those receiving multiple medications with potential metabolic or pharmacokinetic interactions. Depending on the antibacterial agent and the clinical context, relevant monitoring may include serum glucose, sodium, potassium, or thyroid-related parameters. New biochemical abnormalities that develop during treatment should be interpreted in relation to the timing of antibacterial exposure, concomitant medications, organ dysfunction, and the underlying infection before assuming a drug-related endocrine effect.

The main weakness of the current literature is therefore not the absence of proposed mechanisms but the scarcity of studies capable of determining their clinical importance. For several antibacterial agents, it remains unclear how often endocrine abnormalities occur, whether they follow a reproducible dose-response relationship, which patients are genuinely at increased risk, and whether biochemical changes translate into clinically meaningful outcomes. Future clinical research should focus on prospectively defined endocrine endpoints, careful characterization of concomitant treatments and organ dysfunction, and separation of drug-related effects from abnormalities caused by infection or critical illness itself. Such studies would also help determine whether selective laboratory monitoring during antibacterial therapy is justified and, if so, which patient groups would benefit most.

## 10. Limitations

This review has several limitations. Its narrative design does not ensure exhaustive identification of all relevant publications and may be associated with selection bias. The available evidence is highly heterogeneous and includes observational studies, pharmacovigilance analyses, case reports, and experimental models, which limits direct comparison between studies and precludes formal quantitative synthesis or evidence grading. For several antibacterial agents, the available clinical evidence is limited to isolated reports, while pharmacovigilance data are susceptible to reporting bias and cannot establish incidence or causality. In addition, endocrine and metabolic abnormalities observed during antibacterial therapy may be influenced by infection severity, renal dysfunction, nutritional status, concomitant medications, and pre-existing endocrine disorders. Finally, findings from animal, in vitro, and ex vivo models may not directly reflect the effects of standard therapeutic exposure in humans.

## 11. Conclusions

Antibacterial therapy has been associated with a range of endocrine and metabolic disturbances, although their clinical relevance and the nature of the supporting evidence vary substantially between individual agents and outcomes. Reported effects include SIADH-associated hyponatremia, dysglycemia, thyroid-related abnormalities, disturbances of potassium homeostasis, and changes in gonadal hormone profiles. Human clinical and regulatory evidence is most substantial for selected disturbances of glucose and electrolyte homeostasis, whereas many proposed pituitary, thyroid, adrenal, and gonadal effects remain supported primarily by case reports or experimental models. Fluoroquinolone-associated dysglycemia is recognized clinically, while reported reproductive effects remain predominantly experimental. Selected macrolides may influence glucocorticoid metabolism and KCNJ5-dependent aldosterone synthesis, although these findings also remain largely experimental. Linezolid has been associated with hyponatremia and hypoglycemia, while trimethoprim–sulfamethoxazole may contribute to hyponatremia, hyperkalemia, hypoglycemia, and thyroid dysfunction. Older age, renal impairment, polypharmacy, and critical illness may increase susceptibility to some of these complications. Further prospective clinical studies are needed to clarify their incidence, causality, susceptible patient populations, and the potential value of targeted biochemical monitoring.

## Figures and Tables

**Table 1:** Summary of the potential endocrine effects of selected antibacterial agents, the type/source of supporting evidence, and practical clinical considerations.

Endocrine Domain	Antibiotic(s)	Associated Condition/Reported Effect or Proposed Mechanism	Type/Source of Evidence	Clinical Relevance/Practical Considerations	References
Pituitary function	Cefpodoxime	Hyperprolactinemia and galactorrhea	Case reports	Rare case-based association; consider prolactin assessment if compatible symptoms develop.	[28,29]
	Metronidazole	Model-dependent alterations in POMC and prolactin expression; inhibition of ACTH, GH, and PRL secretion in cultured rat pituitary cells	Animal and in vitro studies	Experimental only; no routine endocrine monitoring supported.	[62,63]
Sodium and water homeostasis	Ciprofloxacin	Rarely reported SIADH and hyponatremia	Case reports	Rare case reports; consider serum sodium if compatible symptoms occur or in high-risk patients.	[37,38,39]
	Linezolid	Hyponatremia and SIADH	Clinical and pharmacovigilance data	Clinically relevant in susceptible patients; consider serum sodium monitoring during prolonged therapy or in high-risk patients.	[4,6,57,58]
	Trimethoprim–sulfamethoxazole	SIADH reported in individual cases; trimethoprim may also promote renal sodium loss through an amiloride-like effect	Case reports and mechanistic data	Monitor sodium when clinically indicated, particularly in patients with renal impairment or other risk factors.	[7,68,73]
Glucose homeostasis	Cefditoren	Hypoglycemia; pivalate-associated carnitine depletion may impair fatty acid oxidation and gluconeogenesis	Pharmacovigilance, mechanistic, and case-based evidence	Potential risk in susceptible patients; consider glucose monitoring if symptoms or metabolic risk factors are present.	[11,31,32]
	Fluoroquinolones	Dysglycemia; some agents may stimulate insulin secretion through inhibition of pancreatic β-cell KATP channels	Clinical, pharmacovigilance, and experimental data	Recognized clinical safety issue; consider glucose monitoring in older patients, patients with diabetes, renal impairment, or glucose-lowering therapy.	[11,42,43,44,45]
	Clarithromycin	Increased risk of hypoglycemia, particularly through interactions with repaglinide or sulfonylureas	Case reports and pharmacovigilance data	Review concomitant repaglinide/sulfonylurea therapy; glucose monitoring may be appropriate in at-risk patients.	[11,48,49,50]
	Linezolid	Hypoglycemia, particularly in patients receiving insulin or oral glucose-lowering agents; mechanism remains uncertain	Post-marketing and clinical reports	Consider glucose monitoring in patients receiving insulin or oral glucose-lowering agents.	[59,60,61]
	Trimethoprim–sulfamethoxazole	Hypoglycemia probably related to a sulfonylurea-like effect, particularly in patients with renal impairment	Case reports and case-series analysis	Higher relevance in renal impairment or malnutrition; consider glucose monitoring in susceptible patients.	[71]
Thyroid axis	Cefuroxime and ceftazidime	Inhibition of type 2 deiodinase and reduced local T4-to-T3 conversion	Experimental studies	Experimental evidence; routine thyroid monitoring not supported.	[30]
	Ciprofloxacin	Reduced gastrointestinal absorption of levothyroxine	Drug-interaction data	Clinically relevant drug interaction; separate administration and consider thyroid-function reassessment when clinically indicated.	[41]
	Trimethoprim–sulfamethoxazole	Decreased T4, increased TSH, and thyroid histological changes at prolonged or high-dose exposure	Animal studies	Predominantly animal evidence; routine thyroid monitoring not supported during standard short-course therapy.	[69,70]
Adrenal and HPA axis	Clarithromycin, azithromycin, and roxithromycin	Modulation of local glucocorticoid metabolism and HPA-axis activity	Experimental studies	Experimental only; no routine monitoring supported.	[51,52]
Aldosterone regulation	Clarithromycin and roxithromycin	Selective inhibition of mutant KCNJ5 channels and reduced aldosterone synthesis in KCNJ5-mutated aldosterone-producing adenoma models	In vitro and ex vivo studies	Experimental therapeutic concept rather than an established adverse effect.	[53,54]
Potassium and aldosterone-related homeostasis	Trimethoprim	ENaC inhibition in the distal nephron, reduced potassium secretion, hyperkalemia, and a functional hypoaldosteronism-like state	Mechanistic and clinical data	Clinically relevant renal effect; consider potassium monitoring in patients with renal impairment or other hyperkalemia risk factors.	[72,73]
Gonadal function	Cefuroxime	Reversible reduction in testosterone reported in an experimental model	Experimental evidence summarized in a review	Experimental only; no routine monitoring supported.	[33]
	Clarithromycin + quinestrol	Potentiation of quinestrol-associated suppression of testosterone and LH and reduction in sperm density; likely a pharmacokinetic interaction rather than a direct gonadotoxic effect of clarithromycin	Animal study/pharmacokinetic interaction	Experimental pharmacokinetic interaction; no established relevance to routine antibacterial therapy.	[56]
	Imipenem	Decreased testosterone	Animal studies	Experimental only; no routine monitoring supported.	[33,34]
	Norfloxacin, ciprofloxacin, and enrofloxacin	Alterations in testosterone, progesterone, LH, and FSH	Animal studies	Experimental only; clinical significance in humans remains uncertain.	[46,47]
	Metronidazole	Decreased testosterone, FSH, and LH	Animal studies	Experimental only; no routine monitoring supported.	[65,66]

## Data Availability

No new data were created or analyzed in this study. Data sharing is not applicable to this article.

## Abbreviations

The following abbreviations are used in this manuscript:

ACTH	adrenocorticotropic hormone
ADH	antidiuretic hormone
ADRs	adverse drug reactions
CRH	corticotropin-releasing hormone
CYP3A4	cytochrome P450 3A4
CYP11B2	cytochrome P450 family 11 subfamily B member 2
D1	type 1 iodothyronine deiodinase
D2	type 2 iodothyronine deiodinase
D3	type 3 iodothyronine deiodinase
DHEA	dehydroepiandrosterone
ENaC	epithelial sodium channel
FAERS	FDA Adverse Event Reporting System
FDA	Food and Drug Administration
FQs	fluoroquinolones
FSH	follicle-stimulating hormone
GABA	gamma-aminobutyric acid
GH	growth hormone
GHRH	growth hormone-releasing hormone
GnRH	gonadotropin-releasing hormone
HPA	hypothalamic–pituitary–adrenal
KATP	ATP-sensitive potassium channel
KCNJ5	potassium inwardly rectifying channel subfamily J member 5
LH	luteinizing hormone
MAO	monoamine oxidase
POMC	proopiomelanocortin
PRL	prolactin
PTH	parathyroid hormone
SIADH	syndrome of inappropriate antidiuretic hormone secretion
T3	triiodothyronine
T4	thyroxine
TMP-SMX	trimethoprim–sulfamethoxazole
TRH	thyrotropin-releasing hormone
TSH	thyroid-stimulating hormone
11β-HSD1	11β-hydroxysteroid dehydrogenase type 1
11β-HSD2	11β-hydroxysteroid dehydrogenase type 2
